# Meta-Analysis on the Impact of Inflammatory Rheumatological Conditions on Outcomes Following Acute Coronary Syndrome

**DOI:** 10.7759/cureus.49376

**Published:** 2023-11-24

**Authors:** Marah M Omer, Morshed Alam, Anurag Rawat, Fahad Lakhdhir, Mohammad Alhneif, Dhaval Rabadia, Calvin R Wei, Shamsha Hirani

**Affiliations:** 1 Medicine, Hamad Medical Corporation, Doha, QAT; 2 Internal Medicine, Chittagong Medical College, Chittagong, BGD; 3 Interventional Cardiology, Himalayan Institute of Medical Sciences, Dehradun, IND; 4 Adult Cardiology, National Institute of Cardiovascular Diseases, Karachi, PAK; 5 Cardiology, University of Texas, Texas, USA; 6 Medicine, Surat Municipal Institute of Medical Education and Research, Surat, IND; 7 Research and Development, Shing Huei Group, Taipei, TWN; 8 Cardiology, Baqai Hospital, Karachi, PAK

**Keywords:** meta-analysis, mortality, outcomes, inflammatory rheumatological conditions, acute coronary syndrome

## Abstract

Inflammatory rheumatological conditions, also known as inflammatory rheumatic conditions (IRC), constitute a category of autoimmune and inflammatory ailments primarily affecting the musculoskeletal system, encompassing the joints, muscles, and connective tissues. The objective of this meta-analysis is to evaluate the impact of inflammatory rheumatological conditions (IRC) on post-acute coronary syndrome (ACS) outcomes. This study was performed as per the preferred reporting items for systematic review and meta-analysis (PRISMA) guidelines. The PubMed, Web of Science, and Scopus databases were searched by two authors without any language constraints from January 1, 2015, to October 15, 2023. The primary outcome assessed in this meta-analysis was all-cause mortality. Other outcomes included myocardial infarction and revascularization. A total of 11 studies were included in this meta-analysis. The risk of all-cause mortality was significantly higher in patients with IRC compared to non-IRC patients (RR: 1.12, 95% CI: 1.00 to 1.26, p-value: 0.04). There is a significantly higher risk of myocardial infarction and revascularization in patients with IRC as opposed to those without IRC. Furthermore, while there was a higher risk of stroke in the IRC group compared to the non-IRC group, this disparity did not reach statistical significance. Future research should focus on specific inflammatory rheumatoid conditions, a comprehensive evaluation of cardiovascular events, and targeted interventions to enhance patient outcomes in this vulnerable population.

## Introduction and background

Over the past two decades, a significant medical breakthrough has emerged, revealing that the immune system and inflammatory processes play a role in a broad spectrum of both physical and mental health conditions, which now represent the dominant contributors to global morbidity and mortality [1,2]. In fact, chronic inflammatory diseases have risen to prominence as the leading causes of death worldwide, accounting for over 50% of all fatalities. These conditions encompass inflammation-related maladies such as ischemic heart disease, stroke, cancer, diabetes mellitus, chronic kidney disease, non-alcoholic fatty liver disease (NAFLD), as well as autoimmune and neurodegenerative disorders [3].

Inflammatory rheumatological conditions, also known as inflammatory rheumatic diseases, constitute a category of autoimmune and inflammatory ailments primarily affecting the musculoskeletal system, encompassing the joints, muscles, and connective tissues. These disorders are characterized by persistent inflammation within the affected tissues, resulting in symptoms including pain, swelling, stiffness, and diminished mobility [4]. Notably, these conditions impose a substantial burden on individuals, marked by long-term disabling illnesses, increased mortality rates, and the substantial financial expenses associated with treatment and care [5].

Prior research has demonstrated that individuals afflicted with inflammatory rheumatological conditions, such as rheumatoid arthritis (RA) and systemic lupus erythematosus (SLE), face an elevated risk of experiencing cardiovascular events, including myocardial infarction (MI) [6,7]. Moreover, when these patients develop coronary artery disease, including acute MI, it has been observed that their subsequent clinical outcomes are less favorable compared to those without such conditions [8,9]. This indicates that the presence of inflammatory rheumatological conditions may exacerbate the severity of coronary artery disease and its associated complications [3]. The persistent systemic inflammation inherent in these conditions can foster a proinflammatory environment, potentially hastening atherosclerosis and compromising overall cardiac well-being. It underscores the critical need for vigilant monitoring and tailored treatment approaches for individuals contending with both chronic systemic inflammatory diseases (CSIDs) and coronary artery disease [4].

Given the limited available data regarding acute coronary syndrome (ACS) in patients with inflammatory rheumatological conditions, our objective is to perform a comprehensive meta-analysis. We intend to amalgamate existing studies to compare post-ACS outcomes in individuals with inflammatory rheumatological conditions to those without, thus elucidating the influence of these conditions on ACS prognosis and informing more effective strategies for patient management. The objective of this meta-analysis is to evaluate the impact of inflammatory rheumatological conditions on post-ACS outcomes.

## Review

Methodology

This study was performed as per the preferred reporting items for systematic review and meta-analysis (PRISMA) guidelines.

Literature Search

The PubMed, Web of Science, and Scopus databases were searched by two authors without any language constraints from January 1, 2015, to October 15, 2023. To find additional studies relevant to the study topic, Google Scholar was manually searched. Additionally, forward and backward citation searches of all included articles were performed to find additional relevant studies. Key terms used to search for relevant studies included “inflammatory rheumatological conditions”, “acute coronary syndrome” and “outcomes”. Besides this, their synonyms were used along with medical subject heading (MeSH) terms. Any disagreement between two authors in the process of the literature search was resolved through discussion.

Study Selection

Studies identified through online database searching were exported to EndNote X9 software, and any duplicates were removed. Two investigators independently screened each record title/abstract using the pre-defined inclusion and exclusion criteria. The same two investigators then evaluated the full texts of all records that passed the initial screening process. Any disagreements were resolved through discussion. In the present meta-analysis, we included only those studies that compared the post-ACS outcomes between IRC and non-IRC patients, irrespective of the type of ACS, sample size, geographical region, or study design. We excluded studies that included patients other than ACS. We also excluded reviews, editorials, expert opinions, case reports, and case series.

Data Extraction

Two investigators independently performed the extraction of data using a pre-designed data extraction form designed in Microsoft Office Excel (Redmond, USA). The following data were extracted from the included studies: 1) the characteristics of the study, including author name and publication year. Country of study and sample size; 2) characteristics of participants, including age, gender, diabetes, and hypertension; 3) the outcomes assessed in the present study. All the extracted data was double-checked by two other investigators. The primary outcome assessed in this meta-analysis was all-cause mortality. Other outcomes included myocardial infarction, revascularization, and stroke.

Quality Assessment

The assessment of study quality was carried out meticulously by two independent authors using the Newcastle-Ottawa Scale (NCOS). This robust and widely accepted tool allowed for a comprehensive evaluation of the included studies. The NCOS assesses the quality of non-randomized studies by considering factors such as participant selection, comparability, and outcome assessment, ensuring that a rigorous and standardized approach was employed to ascertain the reliability and validity of the data included in our analysis.

Statistical Analysis

Data analysis was performed using RevMan 5.4.1 (The Cochrane Collaboration, Oxford, United Kingdom). To assess the differences in outcomes between two groups, we calculated the risk ratio (RR) and determined the accompanying 95% confidence interval (CI). Significance was established at a p-value of less than 0.05, indicating statistical significance. To gauge the degree of heterogeneity among the findings from the various studies included in our analysis, we computed the I-square statistic. This measurement was instrumental in evaluating the extent of heterogeneity in the data. A higher I-square value (>50%) signifies significant heterogeneity among the study results.

Results

**Figure 1:**
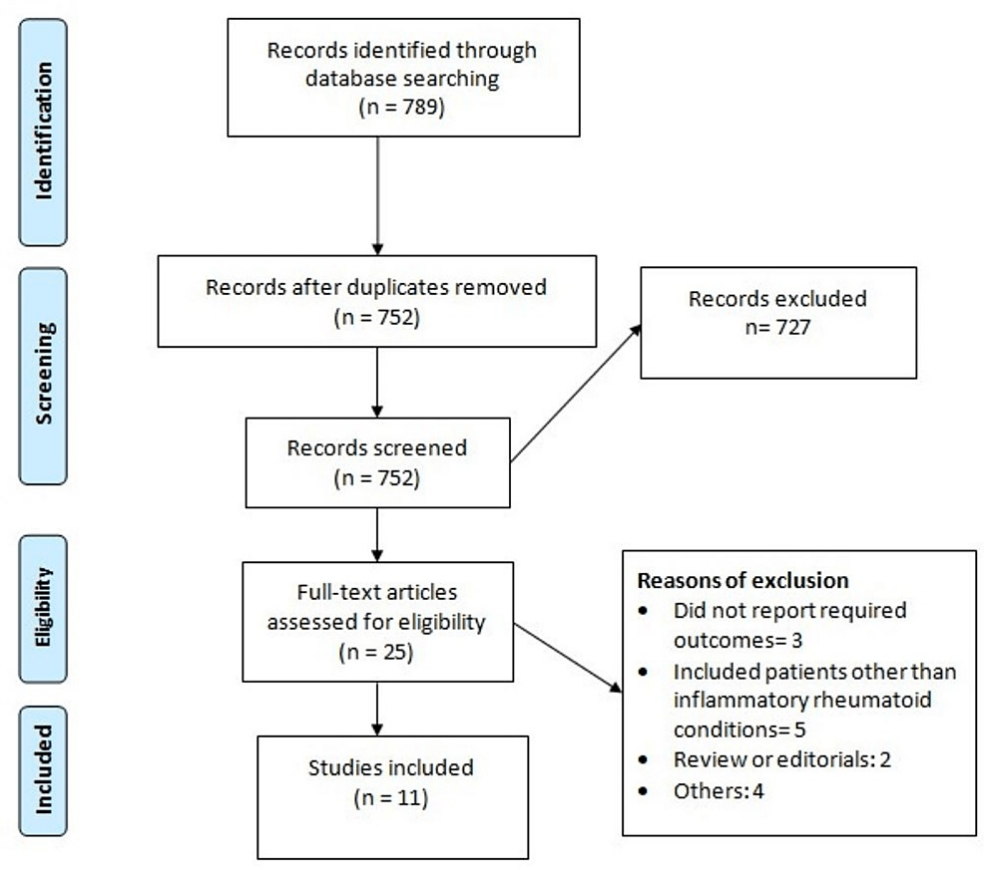
PRISMA flowchart of study selection process

Figure 1 shows the study's selection process. Online database searching yielded 789 records. After removing duplicate studies, the initial screening of 752 studies was done. A full text of 25 records was obtained, and a detailed evaluation was performed using pre-defined inclusion and exclusion criteria. Finally, 11 studies were included in this meta-analysis. Table 1 shows the characteristics of the included studies. The majority of the studies (n=5) were conducted in the United States, while two studies were performed in Sweden. Table 2 presents the quality assessment of the included studies.

**Table 1:** Characteristics of included studies NR: Not reported; NS: Not specified; IRC: Inflammatory rheumatoid condition

Author Name	Year	Region	Disease	Presence of IRC	Sample Size	Mean Age	Males (n)	Diabetes (n)	Hypertension (n)
Ando et al. [10]	2018	United States	Systemic lupus erythematosus	Yes	822	58.88	190	165	518
				No	822	58.72	171	184	534
Desai et al. [11]	2020	United States	Rheumatoid arthritis	Yes	46785	NR	NR	NR	NR
				No	2982910				
Doornum et al. [12]	2015	Australia	NS	Yes	1409	77	515	300	579
				No	77981	74	47492	16183	34687
Elbadawi et al. [13]	2020	United States	Rheumatoid arthritis	Yes	123783	70.41	46312	37244	84447
				No	9235763	67.46	5629014	3161454	6010243
Miyachi et al. [14]	2022	Japan	Psoriasis	Yes	455	66	390	153	319
				No	4459	70	3858	1398	2975
Palomaki et al. [15]	2021	Finland	Rheumatoid arthritis	Yes	1614	73.6	663	364	90
				No	8070	73.9	3253	1856	4561
Skielta et al. [16]	2020	Sweden	Rheumatoid arthritis	Yes	4268	71.05	1895	862	2040
				No	241109	71.07	154069	55455	112357
Sodergren et al. [17]	2021	Sweden	Ankylosing spondylitis	Yes	292	67.9	246	55	157
				No	1276	68.1	1103	172	402
Sokhal et al. [18]	2022	United Kingdom	Polymyalgia Rheumatica	Yes	22597	82	7773	6666	16518
				No	7599445	67	4552068	2606610	5091628
Wassif et al. [19]	2022	United States	NS	Yes	59820	77.09	NR	NR	NR
				No	178547	77.09			
Weber et al. [20]	2022	United States	NS	Yes	53	46	34	11	33
				No	2044	46	1659	405	947

**Table 2:** Quality assessment of included studies

Author Name	Selection	Comparison	Outcome	Overall
Ando et al. [10]	3	2	3	Good
Desai et al. [11]	2	2	3	Good
Doornum et al. [12]	2	2	4	Good
Elbadawi et al. [13]	3	1	3	Good
Miyachi et al. [14]	3	2	3	Good
Palomaki et al. [15]	3	2	4	Good
Skielta et al. [16]	3	1	3	Good
Sodergren et al. [17]	2	2	2	Fair
Sokhal et al. [18]	3	2	3	Good
Wassif et al. [19]	2	1	3	Fair
Weber et al. [20]	2	2	2	Fair

Meta-Analysis of Outcomes

**Figure 2:**
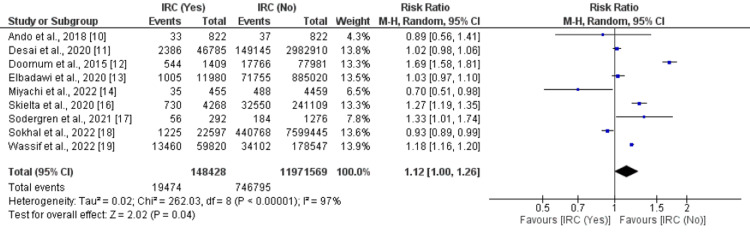
Comparison of all-cause mortality IRC: Inflammatory rheumatoid conditions Sources: References [10-14,16-19]

All-cause mortality: Nine studies were included in the pooled analysis comparing all-cause mortality between IRC and non-IRC groups. As shown in Figure 2, the risk of all-cause mortality was significantly higher in patients with IRC compared to non-IRC patients (RR: 1.12, 95% CI: 1.00 to 1.26, p-value: 0.04). Significant heterogeneity was reported among the study results (I-square: 97%).

Other outcomes: Table 3 presents a comparative analysis of secondary outcomes among patients with and without IRC. The results displayed in Table 3 reveal a significantly elevated risk of myocardial infarction and revascularization in patients with IRC as opposed to those without. Furthermore, while there was a higher risk of stroke in the IRC group compared to the non-IRC group, this disparity did not reach statistical significance (RR: 1.19, 95% CI: 0.92 to 1.55).

**Table 3:** Comparison of secondary outcomes RR: Risk ratio; CI: Confidence interval * Significant at p-value<0.05

Outcome	RR (95% CI)	P-value	I-square
Stroke	1.19 (0.92 to 1.55)	0.19	97%
Myocardial Infarction	1.11 (1.09 to 1.14)*	0.001	0%
Revascularization	1.14 (1.01 to 1.30)*	0.04	88%

Discussion

The objective of this meta-analysis was to assess the outcomes of patients with inflammatory rheumatoid condition (IRC) in comparison to non-IRC individuals following an acute coronary syndrome (ACS). To the best of our knowledge, this represents the inaugural comprehensive meta-analysis that investigates the outcomes of IRC patients versus non-IRC patients after an ACS event. The findings from this meta-analysis indicate a significantly elevated risk of all-cause mortality, recurrent myocardial infarction, and revascularization in IRC patients following ACS. Furthermore, the analysis also reveals a heightened risk of stroke among patients when compared to their non-IRC counterparts, though this disparity did not reach statistical significance.

In individuals experiencing acute myocardial infarction (MI), inflammation may play a crucial role in the progression of coronary atherosclerosis and the occurrence of subsequent events [21]. Experimental investigations have demonstrated the pivotal involvement of inflammation in the development of atherosclerosis in humans. The inflammatory pathogenesis of atherosclerosis encompasses various populations of immune cells, including macrophages and neutrophils, as well as non-immune cells like endothelial and smooth muscle cells [22]. In a clinical context, a Spanish cohort study involving individuals without a history of cardiovascular disease at baseline (n = 991,546) revealed that participants with chronic immune-mediated inflammatory conditions such as rheumatoid arthritis, systemic lupus erythematosus, and inflammatory bowel disease had a significantly higher risk of incident cardiovascular disease over a six-year follow-up period compared to those without such conditions [7].

Considering the elevated risks of unfavorable outcomes observed in patients with IRC, as outlined in the current study, there is a pressing need for additional clinical investigations and therapeutic strategies tailored to this specific patient subgroup. A multicenter prospective study conducted in Switzerland underscored the independent predictive value of systemic inflammation in patients with acute coronary syndrome for adverse cardiovascular outcomes at the one-year mark [23]. Notably, the influential CANTOS trial established the efficacy of anti-inflammatory therapy targeting the interleukin-1β innate immunity pathway in reducing recurrent cardiovascular events in individuals with a history of myocardial infarction [24]. Consequently, anti-inflammatory interventions may hold promise as a therapeutic approach for patients with myocardial infarction and IRC, especially given the findings of the aforementioned French registry suggesting a potential survival benefit associated with corticosteroid use in IRC patients following acute myocardial infarction [22]. However, it is essential to acknowledge that some reports have indicated an increased risk of incident cardiovascular diseases in patients with IRCs, potentially attributed to the promotion of atherosclerosis as a side effect of steroids on hypertension, diabetes, and dyslipidemia [25].

The majority of studies incorporated into this comprehensive meta-analysis have consistently reported unfavorable outcomes among patients diagnosed with IRCs following an episode of acute coronary syndrome. It is worth noting, however, that the specific IRC that exerts the most profound influence on adverse patient outcomes remains undetermined. This uncertainty arises from the fact that no subgroup analysis has been conducted, primarily due to the unavailability of individual-level data within the compiled studies. In light of this knowledge gap, it becomes imperative for future research endeavors to address this critical aspect of IRC-associated acute coronary syndrome. The prioritization of treatment strategies must align with the distinctive needs of patients, emphasizing those who are most susceptible to unfavorable outcomes. Therefore, it is incumbent upon forthcoming studies to delve into this crucial dimension, facilitating a more precise understanding of the interplay between specific IRCs and their impact on patient prognosis, thereby enhancing the efficacy of clinical interventions and ultimately improving patient care.

Study limitations

The current meta-analysis is accompanied by several noteworthy limitations that warrant careful consideration. First, a substantial level of heterogeneity was observed across the analyzed outcomes, potentially attributable to disparities in sample size, variations in the characteristics of the study populations, diverse follow-up durations, and regional influences. Unfortunately, the unavailability of individual-level data precluded the execution of subgroup analyses aimed at elucidating these sources of heterogeneity. Second, it's crucial to underscore that the assessment of revascularization necessity and recurrent myocardial infarction was constrained by a relatively limited number of studies. Hence, it is imperative that forthcoming investigations prioritize these specific endpoints alongside a comprehensive evaluation of various cardiovascular events subsequent to acute coronary syndrome in the context of IRCs. Lastly, all studies included in this meta-analysis adopted an observational design, rendering them susceptible to potential confounding factors.

## Conclusions

In conclusion, this meta-analysis, encompassing 14 studies, demonstrates a significant increase in the risk of all-cause mortality, recurrent myocardial infarction, and revascularization in individuals with inflammatory rheumatoid conditions following acute coronary syndrome. Notably, this pioneering study highlights the crucial role of inflammation in coronary atherosclerosis progression. While anti-inflammatory interventions show promise, caution is warranted due to the potential cardiovascular risks associated with certain treatments. Future research should focus on specific inflammatory rheumatoid conditions, a comprehensive evaluation of cardiovascular events, and targeted interventions to enhance patient outcomes in this vulnerable population.
